# Drivers of prognosis and clinical trajectories differ between COVID and non-COVID acute hypoxic respiratory failure

**DOI:** 10.1371/journal.pone.0339604

**Published:** 2025-12-26

**Authors:** Shaun Pienkos, Andrew R. Moore, Jonasel Roque, Alexandria Jensen, Ana Pacheco-Navarro, Katherine M. Lebold, Caitlin Parmer-Chow, Pablo A. Sanchez, Haley Morin, Christian O’Donnell, Tara Ramaswamy, William Collins, Jennifer G. Wilson, Angela J. Rogers, Joseph E. Levitt

**Affiliations:** 1 Pulmonary and Critical Care Medicine, Stanford University, Stanford, California, United States of America; 2 Stanford University, Stanford, California, United States of America; 3 Division of Pulmonary, Allergy and Critical Care Medicine, Oregon Health & Science University, Portland, Oregon, United States of America; 4 Division of Hospital Medicine, Stanford University, Stanford, California, United States of America; 5 Critical Care Medicine, Stanford, California, United States of America; Wingate University, UNITED STATES OF AMERICA

## Abstract

**Purpose:**

Examine non-respiratory comorbidities that may affect prognosis in acute hypoxic respiratory failure (AHRF) and respiratory trajectories, comparing those with COVID and non-COVID etiologies of AHRF.

**Methods:**

This is a retrospective cohort study of patients with AHRF from COVID and non-COVID etiologies treated with high flow oxygen, noninvasive ventilation, or endotracheal intubation in ICUs in two United States hospitals.

**Results:**

We compared drivers of prognosis and respiratory trajectories between 241 patients with AHRF from COVID and 99 patients with non-COVID AHRF. Patients with COVID had a lower prevalence of major comorbidities or terminal illness (OR 0.14), neurologic disease (OR 0.19), goals of care limitations (OR 0.54), and shock (OR 0.11). A lower proportion of the COVID group were managed with invasive mechanical ventilation (IMV) early in their AHRF course (OR 0.15); however, fewer COVID patients had improvement in AHRF in the first 7 days (OR 0.49), and a greater proportion of COVID patients required IMV on day 14 (OR 2.57). Additionally, fewer COVID patients died or transitioned to comfort care within 14 days following AHRF onset (OR 0.24), and more COVID patients had severe hypoxemia at end-of-life (OR 2.42).

**Conclusions:**

Patients with AHRF from COVID had fewer non-respiratory comorbidities or goals of care limitations, more prolonged respiratory failure and higher risk of mortality related to hypoxemia. These differences could explain why patients with COVID AHRF may experience greater benefit from disease-specific therapies targeting AHRF compared to patients with non-COVID AHRF.

## Introduction

Acute Respiratory Distress Syndrome (ARDS), and Acute Hypoxemic Respiratory Failure (AHRF) more broadly, affect an inherently heterogeneous population due to a significant diversity of etiologies and comorbidities. Despite a high mortality rate (exceeding 30% in many cohorts [1]) which should facilitate well-powered clinical trials, most studies of therapeutics for ARDS prior to the SARS-CoV-2 (COVID) pandemic failed to show benefit [2]. Additionally, interpretation of the few positive trials, such as trials testing corticosteroids [3], is challenging due to similar trials with contradictory findings [4].

The majority of ARDS trials from 2005 to 2015 included patients with conditions associated with significant mortality, such as cancer, organ transplant, immunocompromise, heart failure, and goals of care limitations [5]. A high prevalence of such conditions may contribute to heterogeneity of treatment effect for disease-specific therapies aimed at respiratory recovery, as the risk of death from respiratory failure declines when one’s risk of death from other causes increases [6]. In traditional cohorts of ARDS, most deaths which occur greater than three days after ARDS onset are due to complications of critical illness, not irreversible respiratory failure [7,8], and increasing proportions of ARDS patients die due to withdrawal of life support [9]. Thus, these competing risks for death limit power to detect impact of therapies targeting outcomes such as mortality and ventilator-free days.

Trials of several pharmacologic therapies in COVID, such as the RECOVERY trial testing dexamethasone [10], had few exclusion criteria but nonetheless showed benefit. We suspected that a higher proportion of patients with COVID AHRF died respiratory deaths and had a lower prevalence of competing non-respiratory comorbidities that increase short-term mortality. We further hypothesized that this difference predictively enriched trials of COVID patients (relative to trials in non-COVID ARDS) for patients whose outcomes were more driven by respiratory failure rather than pre-existing comorbidities or goals of care limitations. To test this hypothesis, we compared prevalence of factors with potential to impact prognosis independent of treatment—including goals of care limitations, comorbidities, terminal illness, and shock–along with trajectory and severity of respiratory failure in patients with AHRF with and without COVID. Additionally, we compared the prevalence of severe hypoxemia at time of death between groups.

## Materials and methods

### Patients

We retrospectively examined consecutive patients enrolled into three observational cohorts in two ICUs in the Stanford Healthcare system. Patients enrolled September 2015 – July 2017 in the “Stanford ICU Biorepository” (Stanford IRB #28205) or the “ARDS - Drivers of Prognosis” study (Stanford IRB #68886), and consecutive patients enrolled March 2020 – March 2021 in the “Case analysis of patients with and without COVID-19” study (Stanford IRB #56374) were screened for inclusion. Patients admitted to the ICU with AHRF (defined as receiving high flow nasal oxygen, noninvasive ventilation, or endotracheal intubation), and with oxygen delivery and pulse oximetry data available in the electronic medical record (EMR) were included. Patients were excluded if there was not available data for modified APACHE II score calculation or absent supplemental oxygen delivery and/or pulse oximetry data in the first two days of AHRF. Written informed consent was obtained for all patients for IRB #28205 (recruitment period 10/30/2015–4/24/2017) and a waiver of consent was obtained from the Stanford IRB for protocols #68886 and #56374. All patients in the COVID cohort had a positive RT-PCR for the SARS-CoV-2 virus from nasopharyngeal sampling.

### Evaluation of drivers of prognosis

Each subject was phenotyped by one of 13 clinicians (SP, AJR, PAS, APN, KML, CPC, TR, SG, HM, MJ, JGW, JEL, or ARM) to determine drivers of prognosis by holistic review of the EMR for the first 48 hours after ICU admission. Using a Likert scale (S1 Table), contribution to prognosis at ICU admission was scored from 1–5 (1 being non-contributory and 5 being highly contributory) for the following factors: comorbidities/terminal illness, baseline functional status, and neurologic disease, with a score of four or higher considered a major driver of prognosis. Specifically, vasopressor, goals of care, and respiratory support parameters were extracted from flowsheet data within the EMR, while comorbidities/terminal illness and neurologic disease were obtained from clinician notes. Baseline functional status was determined using notes from case managers, physical therapists, and occupational therapists, who evaluate independence with activities of daily living for patients admitted to the ICU at Stanford. Interrater reliability of the major drivers of prognosis was assessed by two clinicians in 60 patients by percent agreement and Cohen’s kappa. Additionally, we used binary variables for severe shock by vasopressor need (greater than or equal to norepinephrine equivalent of 0.1 microgram/kilogram/minute), severe hypoxemia (oxygen saturation/fraction of inhaled oxygen, or S/F ratio, < 150), and goals of care limitations (any code status other than “full code”) in the first two days after onset of AHRF to designate major contributions from these variables. S/F ratio calculations were limited to oxygen saturation measurements by pulse oximetry which were 97% or lower [11].

For the primary analysis, associations between major drivers of prognosis and in-hospital mortality were compared between patients with and without COVID. Because many ARDS studies also use 28-day mortality as a primary or secondary outcome, we compared in-hospital 28-day mortality between groups using Kaplan Meier estimator and a Cox proportional hazards model which included all described major drivers of prognosis, APACHE II score [12] (modified to exclude pH and with S/F ratio substituted for P/F ratio using a previously described conversion [11] due to a low prevalence of arterial blood gases), and cohort membership as predictor variables. Secondary outcomes included changes in oxygen delivery modes, time to death or transition to comfort care, and severe hypoxemia at end of life or initiation of comfort care.

### Trajectories of respiratory support

For analysis of oxygen delivery modes, we excluded records from 11:00 PM through 6:00 AM to avoid impacts of nocturnal CPAP use. With the remaining oxygen modality records, we identified the most utilized oxygen modality for each patient on each day. Evaluation of respiratory support requirements was performed for 28 days after onset of AHRF to compare trajectories over time. Oxygen delivery modalities and S/F ratios were combined to categorize patients into one of six categories: death or transition to comfort care (Death/CC), invasive mechanical ventilation via endotracheal tube or tracheostomy with S/F < 150 (IMV low S/F), invasive mechanical ventilation via endotracheal tube or tracheostomy with S/F *≥* 150 (IMV high S/F), noninvasive positive pressure ventilation (NIPPV), high flow oxygen via nasal cannula or facemask (HFO), or resolution of supplemental oxygen requirement or low flow oxygen via nasal cannula (resolved). An ordinal scale (resolved, HFO, NIV, IMV high S/F, IMV low S/F, Death/CC) was created for oxygen delivery categories to indicate whether respiratory failure was improving, worsening, or remaining the same.

Study data were collected by EMR review and use of the STAnford Research Repository [13] and managed using REDCap electronic data capture tools hosted at Stanford University [14].

### Statistical analysis

Statistical analyses were performed using R version 4.4.0. Significance was determined at the p < 0.05 level. Comparisons of proportions were evaluated with Fisher’s exact test as implemented by the exact2x2 R package [15], with associated odds ratios and confidence intervals. Comparisons of means were evaluated with the Wilcoxon rank-sum test, and agreements between paired binary variables were assessed by McNemar’s test. The sample size of this cohort size provided a power of >80% to detect a 20% difference in prevalence of drivers of prognosis between the COVID and non-COVID groups.

## Results

### Patient characteristics

Three-hundred eighty-three patients met our criteria for AHRF and were admitted to the ICU. Of these, 17 patients were excluded due to missing data required for calculation of the modified APACHE II score, and 16 more patients were excluded due to missing oxygen modality or S/F ratio data within two days after AHRF onset, leaving a total of 340 patients for analysis. Two-hundred forty-one were hospitalized with acute COVID infection and 99 patients had AHRF due to other causes (Table 1). Notable differences between groups include a higher prevalence of chronic liver disease and prior bone marrow transplantation, and higher modified APACHE II scores in the non-COVID group. Age and sex were similar in both groups, as was prevalence of chronic heart, lung, and renal disease. Primary etiologies of AHRF in the non-COVID cohort included pneumonia (40.4%), non-pulmonary sepsis (26.3%), aspiration (16.2%), trauma (4.0%), transfusion-related acute lung injury (3.0%), pneumonitis (1.0%), pulmonary embolism (1.0%), and organizing pneumonia (1.0%).

**Table 1:** Patient characteristics and early organ function/support. Age, modified APACHE II score, and maximum creatinine are reported as median [interquartile range], and the remainder of characteristics are reported as *n* (percentage of patients). Measures of cardiac, respiratory, and renal function and support through days 0-1 of AHRF are reported.

Patient Characteristic	COVID (n = 241)	Non-COVID (n = 99)	p value
Age	65.0 [51.0, 77.0]	63.0 [47.5, 72.5]	0.16
Female	92 (38.2%)	41 (41.4%)	0.63
Congestive Heart Failure	65 (27.0%)	36 (36.4%)	0.09
Chronic Lung Disease	57 (23.7%)	34 (34.3%)	0.06
Chronic Liver Disease	1 (0.4%)	4 (4.0%)	0.03
End Stage Renal Disease	15 (6.2%)	8 (8.1%)	0.63
Solid Organ Transplant	19 (7.9%)	5 (5.1%)	0.49
Bone Marrow Transplant	3 (1.2%)	11 (11.1%)	<0.001
Modified APACHE II	25.0 [18.0, 32.0]	30.0 [25.0, 38.5]	<0.001
Day 1 S/F < 150	111 (46.1%)	38 (38.4%)	0.23
Vasopressors/Inotropes	76 (31.5%)	78 (78.8%)	<0.001
Maximum Creatinine (mg/dL)	0.94 [0.65, 1.49]	1.29 [0.89, 2.16]	<0.001
Day 1 Respiratory Support			
High Flow Nasal Oxygen	167 (69.3%)	18 (18.2%)	<0.001
Invasive Mechanical Ventilation	69 (28.6%)	71 (71.7%)	
Non-Invasive Ventilation	5 (2.1%)	10 (10.1%)	

### Early organ failure and mortality

COVID patients had lower rates of shock (31.5% versus 78.8%, OR 0.12, 95% CI 0.07–0.22, p < 0.001), intubation (28.6% versus 71.7%, p < 0.001), and lower peak creatinine (0.94 versus 1.29 milligrams/deciliter, p < 0.001) on day one of AHRF (Table 1). While noninvasive ventilation was rarely used in either cohort, high-flow nasal oxygen was more common in COVID patients (69.3% versus 18.2%, p < 0.001). In-hospital mortality was lower in COVID compared to non-COVID patients (29.9% versus 41.4%, OR 0.60, 95% CI 0.37–1.00, p = 0.04).

### Drivers of prognosis

**Fig 1:**
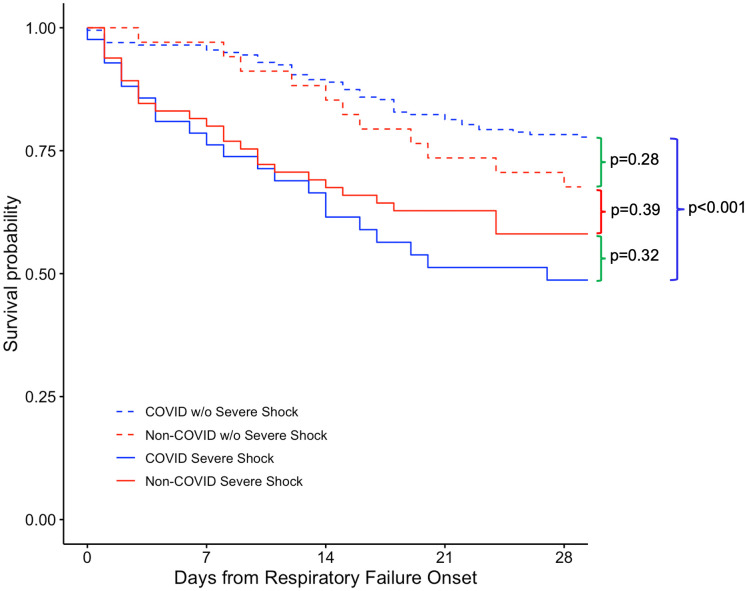
Kaplan-Meier survival analysis of patients stratified by cohort and presence of severe shock. p values shown compare in-hospital 28-day mortality between those with severe shock versus those without severe shock in the COVID and non-COVID cohorts using Fisher’s exact test.

**Fig 2:**
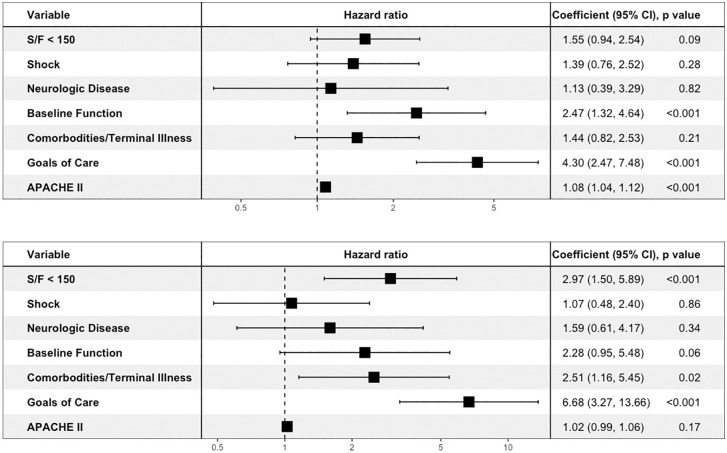
Forest plots of Cox proportional hazards model of 28-day mortality in A) COVID patients and B) non-COVID patients.

Interrater agreement for major drivers of prognosis ranged from 87%−92% with Cohen’s kappas between 0.71–0.73 (except for neurologic disease that had a kappa of zero due to only six patients with this designated as a major driver and a lack of agreement between clinicians on the importance of this variable in these patients). Comparison of major drivers of prognosis in the COVID versus non-COVID cohorts revealed important differences early in the course of AHRF (Table 2). Major comorbidities or terminal illness (17.8% in the COVID group versus 61.6% in the non-COVID group, OR 0.14, 95% CI 0.08–0.24, p < 0.001), neurologic disease (2.5% versus 12.1%, OR 0.19, 95% CI 0.07–0.51, p < 0.001), goals of care limitations (13.3% versus 22.2%, OR 0.54, 95% CI 0.29–1.00, p < 0.05), and shock (17.4% versus 65.7%, OR 0.11, 95% CI 0.06–0.19, p < 0.001) were all less prevalent in the COVID cohort. Baseline functional limitations were less prevalent in COVID patients (9.1% versus 16.2%, OR 0.52, 95% CI 0.25–1.10, p = 0.09), while severe hypoxemia was more common (46.1% versus 38.4%, OR 1.37, 95% CI 0.84–2.24, p = 0.23); however, this difference was not statistically significant. Because of the potential for confounding by the marked differences in prevalence of shock, we stratified by cohort and presence of shock as a major driver of prognosis. In this analysis, shock was associated with significantly higher mortality in patients with COVID but not in non-COVID patients (Fig 1). A Cox proportional hazards model of 28-day mortality in COVID patients identified goals of care limitations, baseline functional limitations, and APACHE II score as statistically significant predictors of mortality (Fig 2A). In non-COVID patients, severe hypoxemia, comorbidities and terminal illness, and goals of care limitations were associated with 28-day mortality (Fig 2B). When both cohorts were combined, COVID versus non-COVID etiology of AHRF was not a significant predictor of mortality in the Cox proportional hazards model.

**Table 2:** Comparing drivers of prognosis in COVID and non-COVID patients. Percentage of patients with each driver of prognosis are reported, with odds ratios and confidence intervals (CI) for Fisher’s exact test results.

Driver of Prognosis	COVID	Non-COVID	Odds Ratio (95% CI)	p value
Respiratory Failure	46.1%	38.4%	1.37 (0.84-2.24)	0.23
Severe Shock	17.4%	65.7%	0.11 (0.06-0.19)	<0.001
Comorbidities	17.8%	61.6%	0.14 (0.08-0.24)	<0.001
Neurologic Disease	2.5%	12.1%	0.19 (0.07-0.51)	<0.001
Baseline Functional Limitations	9.1%	16.2%	0.52 (0.25-1.10)	0.09
Goals of Care Limitations	13.3%	22.2%	0.54 (0.29-1.00)	0.05

### Trajectories of respiratory support

**Fig 3:**
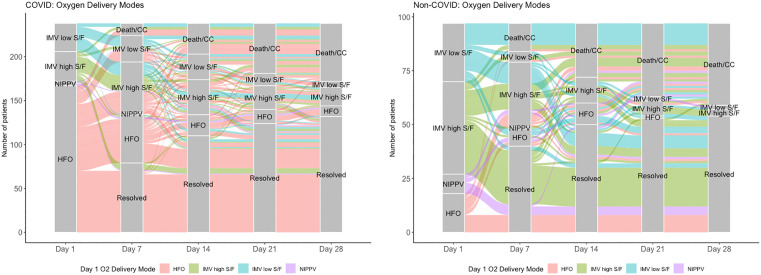
Respiratory support trajectories for A) COVID patients (n = 240) and B) non-COVID patients (n = 97) at days 1, 7, 14, 21, and 28 after onset of AHRF. Death/CC = death or transition to comfort care; IMV low S/F = invasive mechanical ventilation via endotracheal tube or tracheostomy with S/F < 150; IMV high S/F = invasive mechanical ventilation via endotracheal tube or tracheostomy with S/F ≥ 150; NIPPV = noninvasive positive pressure ventilation; HFO = high flow oxygen via nasal cannula or face mask; Resolved = resolution of supplemental oxygen requirement or low flow oxygen via nasal cannula.

Three patients were excluded from this analysis because supplemental oxygen delivery and/or pulse oximetry data was not available for more than three of 28 days. On day 1 of AHRF, a lower proportion of COVID patients received IMV (27.8% versus 72.2% in the non-COVID group, OR 0.15, 95% CI 0.08–0.26, p < 0.001) (Fig 3 A, B). Between day 1 and day 7 of AHRF, a lower proportion of COVID patients improved on our ordinal scale of respiratory support (41.3% versus 58.8%, OR 0.49, 95% CI 0.30–0.82, p = 0.004). Of the patients that required IMV on day 1 of AHRF, a lower percentage in the COVID cohort had died or transitioned to comfort care on day 7 (2.9% versus 12.4%, OR 0.21, 95% CI 0.07–0.61, p = 0.001) and day 14 (5.8% versus 20.6%, OR 0.24, 95% CI 0.11–0.53, p < 0.001). Of the patients who were alive on day 14, a higher percentage of COVID patients required IMV (34.0% versus 16.7%, OR 2.57, 95% CI 1.26–5.60, p = 0.006) (Fig 3 A, B). This prolonged hypoxemia in COVID was not driven by differences in goals of care limitations regarding endotracheal intubation. Among patients without “do not intubate” preferences at AHRF onset, substantially more COVID patients required IMV on day 14 (29.9% versus 12.6%, OR 2.94, 95% CI 1.47–6.30, p = 0.001) (S1A, B Fig).

These differences in duration of hypoxemia are not explained by a higher proportion of patients with COVID meeting criteria on HFO. Among patients receiving IMV on day 1 of AHRF (S2A, B Fig), fewer COVID patients resolved their AHRF by day 7 (12.1% versus 38.6%, OR 0.22, 95% CI 0.09–0.54, p < 0.001) and by day 14 (22.7% versus 50.0%, OR 0.30, 95% CI 0.13–0.63, p = 0.001).

### End-of-life respiratory failure

More COVID patients had severe hypoxemia (S/F < 150) in the two days prior to death or transition to comfort-oriented care (47.2% versus 26.8%, OR 2.42, 95% CI 1.06–5.85, p < 0.05). The mean of the maximum S/F ratio in this time period was 181 in the COVID group and 215 in the non-COVID group (p = 0.02). In both cohorts, presence of severe hypoxemia early in the course of AHRF was correlated to severe hypoxemia at the end of life (p = 0.02).

## Discussion

When comparing drivers of prognosis in patients with COVID and non-COVID AHRF, we found that COVID patients had fewer non-respiratory drivers of a poor prognosis. In addition, patients with COVID had more prolonged respiratory failure and more severe hypoxemia at time of death suggesting their AHRF was a more prominent determinant of their outcome, potentially providing a long therapeutic window for interventions and increasing the likelihood of benefit from disease-specific therapies targeting lung injury.

Specifically, we found that all major drivers of prognosis (goals of care limitations, poor baseline functional status, comorbidities, neurologic status, and severe shock) except severe hypoxemia were more prevalent in the non-COVID cohort. Severe shock was approximately fourfold more prevalent in non-COVID patients. In unadjusted models, shock was not associated with mortality in non-COVID patients but was associated with a roughly threefold increase in mortality in COVID patients. We suspect this is because shock, especially due to bacterial sepsis, is a common underlying cause of AHRF in non-COVID patients and thus may not be as important as an independent driver of prognosis. However, shock is less common in viral sepsis and therefore may serve as a marker of more severe critical illness and secondary infections in AHRF due to COVID. Similar reasoning may explain why severe hypoxemia was associated with higher mortality in non-COVID but not in COVID patients. Since prognosis in COVID was driven more by AHRF, severe hypoxemia was paradoxically a less unique driver of prognosis, while severe hypoxemia was less prevalent in non-COVID patients who were characterized more by shock, and therefore severe hypoxemia identified a higher risk subgroup of non-COVID patients but not in COVID patients. Conversely, the opposite was true regarding the significance of shock in COVID and non-COVID patients. Major comorbidities or terminal illness were also greater than threefold more prevalent in non-COVID patients, and the presence of major comorbidities or terminal illness was associated with an increased risk of death in the non-COVID cohort. This highlights that in unselected populations of critically ill patients with AHRF, other drivers of prognosis may significantly impact outcomes.

We also found important differences in the trajectory of critical illness between patients with and without COVID. First, while severity of hypoxemia, as measured by S/F ratio, was similar between the two cohorts on day one of AHRF, among survivors, fewer patients resolved their AHRF by day 7 and 14 in the COVID group. Second, although a lower proportion of COVID patients required IMV on day 1, a greater proportion required IMV on day 14, suggesting that more patients with COVID experienced progressive and more prolonged respiratory failure relative to patients without COVID. Third, severe hypoxemia within the first 48 hours of ICU admission was not a statistically significant predictor in our adjusted model of 28-day mortality in the COVID cohort, but a higher percentage of patients who died in the COVID group had severe hypoxemia (defined by an S/F < 150) at time of death or transition to comfort care. These results further highlight the importance of progressive and prolonged respiratory failure in addition to severe hypoxemia early in the disease course.

These important differences between COVID patients with AHRF and historic cohorts of non-COVID patients with ARDS likely explain the greater success rate of clinical trials during the COVID pandemic [16,17] relative to previous trials in unselected populations of ARDS. Our results also have important implications for the design of future trials targeting patients with AHRF. First, a high proportion of patients demonstrating rapid improvement in hypoxemia (potentially associated with rapidly improving shock) after onset of AHRF will likely limit the power of trials to detect clinical benefit due to a narrower window for an intervention to impact outcomes relative to recovery among controls with usual care alone. Second, a heavy burden of comorbidities and other non-respiratory drivers of a poor prognosis might increase prognostic enrichment (i.e., prevalence of endpoints) but it will almost certainly reduce predictive enrichment (i.e., probability of a treatment benefit) by introducing greater treatment-independent risk of death or organ failure. Finally, similar to other contemporary analyses of patients with ARDS [18,19], fewer than a third of non-COVID patients had severe hypoxemia at the end of life, suggesting that refractory hypoxemia was not directly responsible for death in most individuals. In contrast, severe hypoxemia at time of death was almost twice as common in patients with COVID. Given that mortality has been the primary endpoint in most trials of AHRF/ARDS, developing strategies to enrich enrollment for patients likely to develop refractory hypoxemia, which is more likely to be a treatment-dependent risk factor for death, will be important for future trials, especially those testing ventilator strategies or other lung-specific interventions. Alternatively, an endpoint of decreasing respiratory support requirements or more rapid resolution of AHRF may be a more statistically powerful while still clinically meaningful endpoint relative to mortality in future trials.

This study has several strengths. First, Likert scales were completed by 13 different clinicians, and categorization of patients had good interrater agreement, increasing the generalizability of our findings. Second, our use of S/F ratios and an ordinal scale of oxygen delivery modalities as indicators of severity and trajectory of lung injury offers a measure which is easily obtainable at frequent intervals and does not require additional laboratory studies. Additionally, use of S/F ratios is in line with recent updates to the ARDS definition, partially aimed at improving applicability to limited-resource settings where arterial blood gases may not be readily available [20]. Lastly, detailed examination of trajectories of severity of AHRF may provide new insights into subgroups that are most likely to benefit from interventions for AHRF.

There are also multiple limitations. First, participants from both cohorts were recruited from a single academic medical system which operates as a regional cancer and transplant center, thus a higher prevalence of malignancy, and solid-organ and hematologic transplant recipients in the non-COVID intensive care population may reduce generalizability to many community hospitals with a lower prevalence of these comorbidities. Second, COVID patients were recruited early in the pandemic, which may limit generalizability to current COVID populations, which are known to have different comorbidities than early pandemic comparators [21–23]. Third, evaluation of several of the drivers of prognosis involved subjective assessments that may limit reproducibility. However, our survival models showed that the subjective elements added additional predictive information to the APACHE II score, suggesting these measures hold important prognostic information. Automated extraction of these data from the EMR using tools such as large language models may be of value for future analyses, but would require prospective validation and was not feasible for this analysis.

Additionally, changes in clinical practice patterns between our COVID and historical non-COVID cohorts (enrolled in 2020–2021 and 2015–2017, respectively) may have affected the observed respiratory trajectories. For example, use of HFO was much more common during the COVID pandemic and more patients with COVID met criteria for AHRF while receiving HFO, which complicates comparison of severity of AHRF by S/F ratio between cohorts given expected effects of positive end-expiratory pressure on S/F ratios. Greater use of HFO may have delayed intubation in patients with COVID [24], which could influence the prevalence and severity of complications such as self-inflicted lung injury and ventilatory-induced lung injury. Delayed intubation has previously been associated with higher mortality in AHRF from COVID [24] and non-COVID etiologies [25,26], though randomized trial data regarding this association are lacking, and practice patterns may have changed between “waves” within the COVID pandemic [24]. Additionally, prone positioning in the awake patient became much more widespread during the COVID pandemic and may impact major outcomes such as endotracheal intubation and mortality [27]. The numerous extrapulmonary factors that can influence timing of intubation (neurologic status, shock, differences in clinical practice patterns, etc.) further complicate interpretation of respiratory trajectories. Importantly, there was no formalized protocol within our medical system for transitioning from one respiratory support modality to another. We have attempted to address these uncertainties by conducting a sensitivity analysis of respiratory trajectories limited to patients requiring IMV at onset of AHRF that yielded similar results. However, our respiratory trajectory findings should still be interpreted with caution due to the aforementioned factors that could not be controlled due to non-contemporaneous cohorts and the observational nature of this study.

Finally, this study does not directly evaluate biologic heterogeneity in AHRF. Multiple approaches exist to categorize patients through transcriptomics or proteomics in non-COVID ARDS cohorts, with evidence of differential treatment response between groups of patients based on these biologic classifications [28]. Future studies may consider evaluating the relationship of drivers of prognosis and biologic classifications, as both factors may prove useful for clinical trial enrichment strategies.

## Conclusions

In conclusion, this study identified significant differences between patients with AHRF due to COVID compared to non-COVID etiologies of AHRF that likely have important implications for clinical trials in these patients. We found a significantly greater burden of non-respiratory drivers of a poor prognosis in non-COVID patients, and more protracted AHRF in patients with COVID with a greater proportion having severe hypoxemia at time of death. We suspect that these differences led to predictive enrichment of clinical trials early in the COVID pandemic and likely contributed to the relative success of pharmacologic interventions in COVID trials compared to trials in non-COVID ARDS. Our findings may have important implications for designing future trials of AHRF in COVID and non-COVID patients.

## Supporting information

S1 Fig Respiratory support trajectories for A) COVID patients (n = 231) and B) non-COVID patients (n = 95) who did not specify “do not intubate” for their initial code status. Death/CC = death or transition to comfort care; IMV low S/F = invasive mechanical ventilation via endotracheal tube or tracheostomy with S/F < 150; IMV high S/F = invasive mechanical ventilation via endotracheal tube or tracheostomy with S/F ≥ 150; NIPPV = noninvasive positive pressure ventilation; HFO = high flow oxygen via nasal cannula or facemask; Resolved = resolution of supplemental oxygen requirement or low flow oxygen via nasal cannula.
(PDF)

S2 Fig Respiratory support trajectories for A) COVID patients (n = 66) and B) non-COVID patients (n = 70) who were receiving invasive mechanical ventilation on day 1 of AHRF. Death/CC = death or transition to comfort care; IMV low S/F = invasive mechanical ventilation via endotracheal tube or tracheostomy with S/F < 150; IMV high S/F = invasive mechanical ventilation via endotracheal tube or tracheostomy with S/F ≥ 150; NIPPV = noninvasive positive pressure ventilation; HFO = high flow oxygen via nasal cannula or facemask; Resolved = resolution of supplemental oxygen requirement or low flow oxygen via nasal cannula.
(PDF)

S1 Table Descriptions used to guide evaluation of drivers of prognosis. Numeric scores and corresponding categorizations are shown in column headers.
(PDF)

S2 Table Cox proportional hazards models for A) COVID and B) non-COVID cohorts. (PDF)
