# Supplementary figures and images for: Drivers of prognosis and clinical trajectories differ between COVID and non-COVID acute hypoxic respiratory failure
Source: PLoS One. 2025 Dec 26;20(12):e0339604. doi: 10.1371/journal.pone.0339604 (PMC12742738; doi:10.1371/journal.pone.0339604)

# COVID: Oxygen Delivery Modes

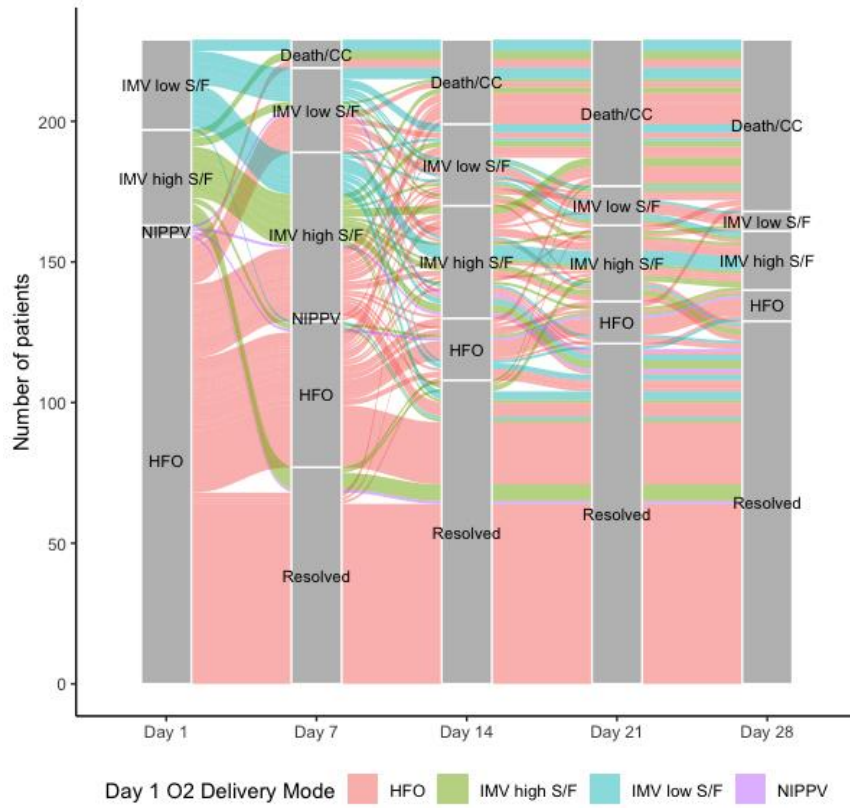

A)

## Non-COVID: Oxygen Delivery Modes

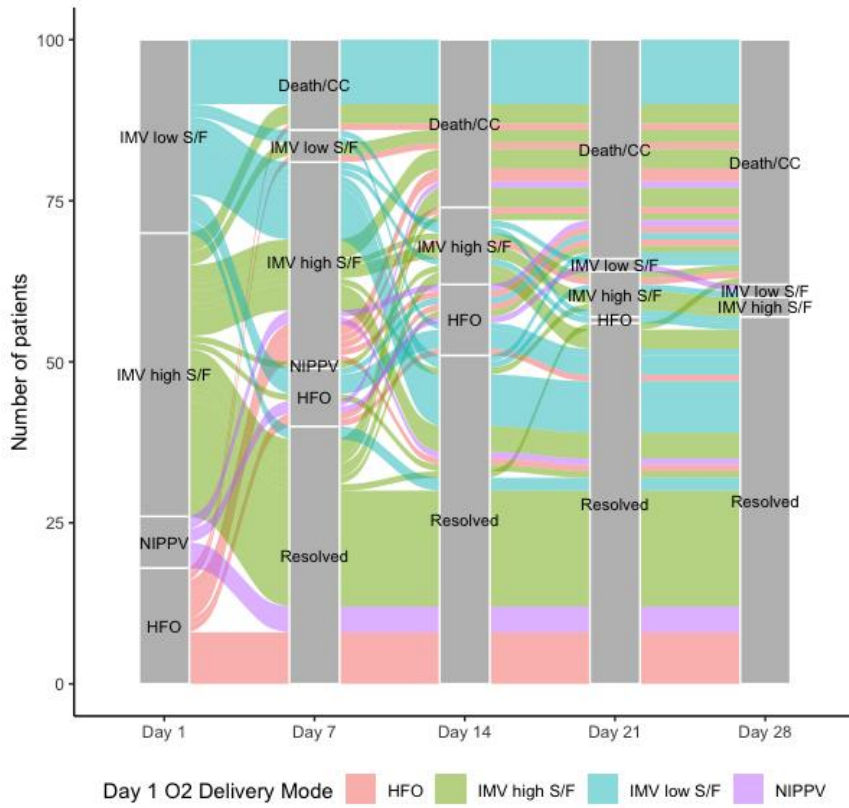

**B)**

Supplement: S1 Fig — Death/CC = death or transition to comfort care; IMV low S/F = invasive mechanical ventilation via endotracheal tube or tracheostomy with S/F < 150; IMV high S/F = invasive mechanical ventilation via endotracheal tube or tracheostomy with S/F ≥ 150; NIPPV = noninvasive positive pressure ventilation; HFO = high flow oxygen via nasal cannula or facemask; Resolved = resolution of supplemental oxygen requirement or low flow oxygen via nasal cannula. (PDF) [file pone.0339604.s001.pdf]
