# Supplementary material for: Drivers of prognosis and clinical trajectories differ between COVID and non-COVID acute hypoxic respiratory failure
Source: PLoS One. 2025 Dec 26;20(12):e0339604. doi: 10.1371/journal.pone.0339604 (PMC12742738; doi:10.1371/journal.pone.0339604)
Supplement: S2 Fig — Death/CC = death or transition to comfort care; IMV low S/F = invasive mechanical ventilation via endotracheal tube or tracheostomy with S/F < 150; IMV high S/F = invasive mechanical ventilation via endotracheal tube or tracheostomy with S/F ≥ 150; NIPPV = noninvasive positive pressure ventilation; HFO = high flow oxygen via nasal cannula or facemask; Resolved = resolution of supplemental oxygen requirement or low flow oxygen via nasal cannula. (PDF) [file pone.0339604.s002.pdf]

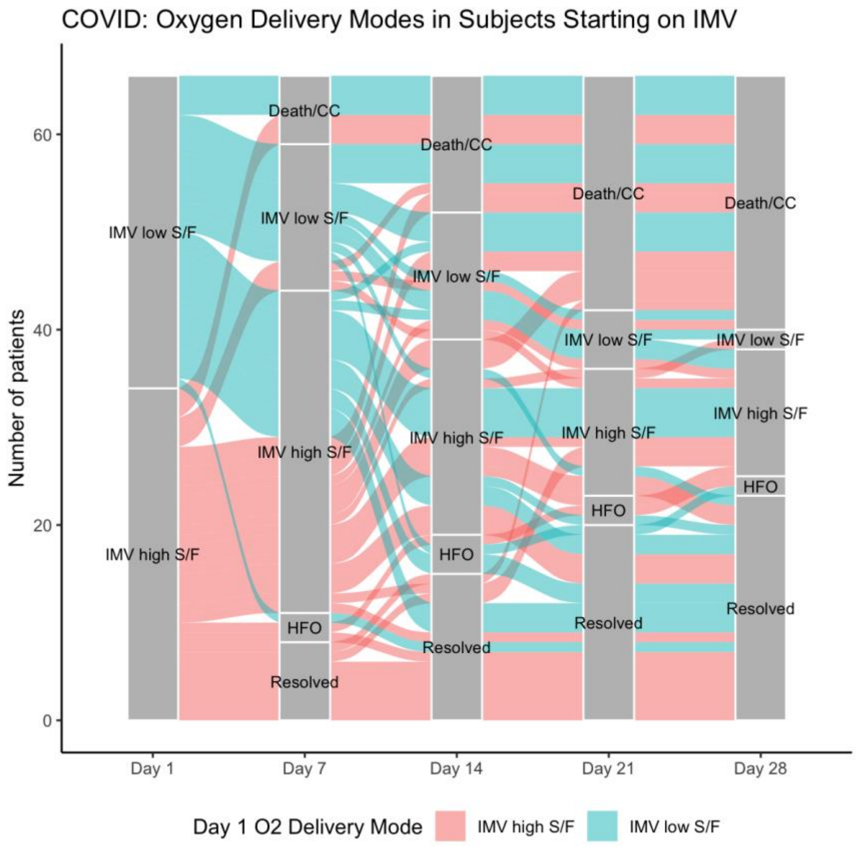

COVID: Oxygen Delivery Modes in Subjects Starting on IMV
Number of patients
0
20
40
60
IMV low S/F
IMV high S/F
Death/CC
IMV low S/F
IMV high S/F
HFO
Resolved
Death/CC
IMV low S/F
IMV high S/F
HFO
Resolved
Death/CC
IMV low S/F
IMV high S/F
HFO
Resolved
Death/CC
IMV low S/F
IMV high S/F
HFO
Resolved
Day 1
Day 7
Day 14
Day 21
Day 28
Day 1 O2 Delivery Mode
IMV high S/F
IMV low S/F

A)

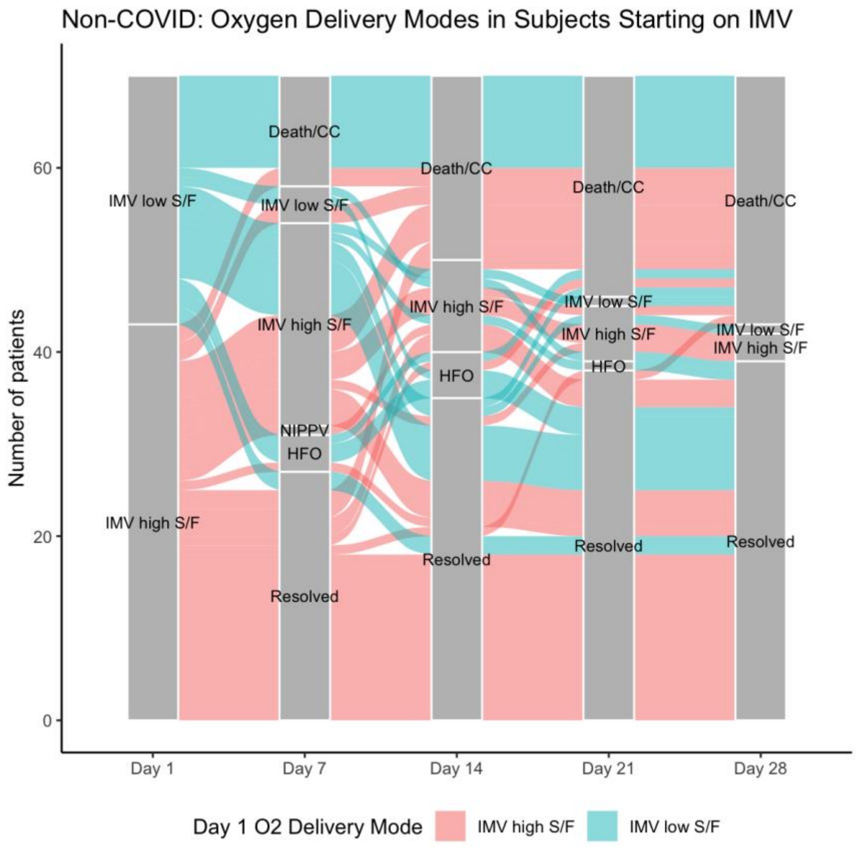
Non-COVID: Oxygen Delivery Modes in Subjects Starting on IMV
Number of patients
0
20
40
60
Day 1
Day 7
Day 14
Day 21
Day 28
IMV low S/F
IMV high S/F
Death/CC
IMV low S/F
IMV high S/F
NIPPV
HFO
Resolved
Death/CC
IMV high S/F
HFO
Resolved
Death/CC
IMV low S/F
IMV high S/F
HFO
Resolved
Death/CC
IMV low S/F
IMV high S/F
Resolved
Day 1 O2 Delivery Mode
IMV high S/F
IMV low S/F

B)
