# Supplementary material for: Drivers of prognosis and clinical trajectories differ between COVID and non-COVID acute hypoxic respiratory failure
Source: PLoS One. 2025 Dec 26;20(12):e0339604. doi: 10.1371/journal.pone.0339604 (PMC12742738; doi:10.1371/journal.pone.0339604)
Supplement: S1 Table — Numeric scores and corresponding categorizations are shown in column headers. (PDF) [file pone.0339604.s003.pdf]

Table S1

Driver of Prognosis	1 (Noncontributory)	2-3 (Minor Driver)	4-5 (Major Driver)
**Comorbidities/Terminal Illness**	No significant comorbidities	Moderate Comorbidities or terminal illness (e.g. uncontrolled diabetes, HTN, advanced CKD, moderate COPD, metastatic castrate-sensitive prostate cancer)	Decompensated organ failure (e.g. heart failure, cirrhosis, ESRD) or terminal illness with poor prognosis (e.g. metastatic cancer causing organ failure)
**Baseline Functional Status**	Independent in activities of daily living (ADL) and instrumental activities of daily living (IADL)	Dependent for some ADLs/IADLs	Severe immobility/lack of independence
**Neurologic Disease**	No significant neurologic disorder	Moderate neurologic disorder (e.g. long-standing active seizure disorder, prior stroke with significant residual dysfunction)	Life-threatening neurologic disorder (e.g. subarachnoid hemorrhage, major stroke, status epilepticus)
**Goals of Care Limitations**	Full Code		Any code status limitation
**Shock**	Norepinephrine equivalent <0.05mcg/kg/min	Norepinephrine equivalent 0.05-0.1 mcg/kg/min	Norepinephrine equivalent >0.1 mcg/kg/min
**Acute Respiratory Failure**	Room air or low-flow nasal oxygen	CPAP/BiPAP/High-flow nasal oxygen/Invasive mechanical ventilation with S:F≥150	CPAP/BiPAP/High-flow nasal oxygen/Invasive mechanical ventilation with S:F<150
