# Supplementary material for: Drivers of prognosis and clinical trajectories differ between COVID and non-COVID acute hypoxic respiratory failure
Source: PLoS One. 2025 Dec 26;20(12):e0339604. doi: 10.1371/journal.pone.0339604 (PMC12742738; doi:10.1371/journal.pone.0339604)
Supplement: S2 Table — (PDF) [file pone.0339604.s004.pdf]

Table S2A: COVID cohort

Variable	coefficient	standard error	lower 95%	upper 95%	p value
`S/F < 150`	1.54636	0.25382	0.9403	2.543	0.0859
Shock	1.38926	0.30476	0.7645	2.525	0.28069
`Neurologic Disease`	1.13242	0.54411	0.3898	3.29	0.81922
`Baseline Function`	2.46935	0.32141	1.3152	4.636	0.00492
`Comorbidities/Terminal Illness`	1.43879	0.28803	0.8181	2.53	0.20656
`Goals of Care`	4.29848	0.2829	2.4689	7.484	2.54E-07
`APACHE II`	1.07881	0.01838	1.0406	1.118	3.68E-05

Concordance= 0.784 (se = 0.029)
Likelihood ratio test= 83.37 on 7 df, p=3e-15
Wald test = 88.2 on 7 df, p=3e-16
Score (logrank) test = 116.9 on 7 df, p=<2e-16

Table S2B: Non-COVID cohort

Variable	coefficient	standard error	lower 95%	upper 95%	p value
`S/F < 150`	2.97348	0.34883	1.5009	5.891	0.00178
Shock	1.0725	0.40996	0.4802	2.395	0.86443
`Neurologic Disease`	1.59406	0.49039	0.6097	4.168	0.34168
`Baseline Function`	2.2834	0.44699	0.9508	5.484	0.06472
`Comorbidities/Terminal Illness`	2.50924	0.39571	1.1553	5.45	0.02008
`Goals of Care`	6.68E+00	0.36464	3.2709	13.659	1.89E-07
`APACHE II`	1.02485	1.79E-02	0.9896	1.061	0.16918

Concordance= 0.755 (se = 0.044)
Likelihood ratio test= 41.54 on 7 df, p=6e-07
Wald test = 38.64 on 7 df, p=2e-06
Score (logrank) test = 48.35 on 7 df, p=3e-08
